# Background Factors of Reflux Esophagitis and Non-Erosive Reflux Disease: A Cross-Sectional Study of 10,837 Subjects in Japan

**DOI:** 10.1371/journal.pone.0069891

**Published:** 2013-07-26

**Authors:** Chihiro Minatsuki, Nobutake Yamamichi, Takeshi Shimamoto, Hikaru Kakimoto, Yu Takahashi, Mitsuhiro Fujishiro, Yoshiki Sakaguchi, Chiemi Nakayama, Maki Konno-Shimizu, Rie Matsuda, Satoshi Mochizuki, Itsuko Asada-Hirayama, Yosuke Tsuji, Shinya Kodashima, Satoshi Ono, Keiko Niimi, Toru Mitsushima, Kazuhiko Koike

**Affiliations:** 1 Department of Gastroenterology, Graduate School of Medicine, The University of Tokyo, Tokyo, Japan; 2 Kameda Medical Center Makuhari, Chiba-shi, Japan; National Cancer Center, Japan

## Abstract

**Background:**

Despite the high prevalence of gastroesophageal reflux disease (GERD), its risk factors are still a subject of controversy. This is probably due to inadequate distinction between reflux esophagitis (RE) and non-erosive reflux disease (NERD), and is also due to inadequate evaluation of adjacent stomach. Our aim is therefore to define background factors of RE and NERD independently, based on the evaluation of *Helicobacter pylori* infection and gastric atrophy.

**Methods:**

We analyzed 10,837 healthy Japanese subjects (6,332 men and 4,505 women, aged 20–87 years) who underwent upper gastrointestinal endoscopy. RE was diagnosed as the presence of mucosal break, and NERD was diagnosed as the presence of heartburn and/or acid regurgitation in RE-free subjects. Using GERD-free subjects as control, background factors for RE and NERD were separately analyzed using logistic regression to evaluate standardized coefficients (SC), odds ratio (OR), and *p*-value.

**Results:**

Of the 10,837 study subjects, we diagnosed 733 (6.8%) as RE and 1,722 (15.9%) as NERD. For RE, male gender (SC = 0.557, OR = 1.75), *HP* non-infection (SC = 0.552, OR = 1.74), higher pepsinogen I/II ratio (SC = 0.496, OR = 1.64), higher BMI (SC = 0.464, OR = 1.60), alcohol drinking (SC = 0.161, OR = 1.17), older age (SC = 0.148, OR = 1.16), and smoking (SC = 0.129, OR = 1.14) are positively correlated factors. For NERD, *HP* infection (SC = 0.106, OR = 1.11), female gender (SC = 0.099, OR = 1.10), younger age (SC = 0.099, OR = 1.10), higher pepsinogen I/II ratio (SC = 0.099, OR = 1.10), smoking (SC = 0.080, OR = 1.08), higher BMI (SC = 0.078, OR = 1.08), and alcohol drinking (SC = 0.076, OR = 1.08) are positively correlated factors. Prevalence of RE in subjects with chronic *HP* infection and successful *HP* eradication denotes significant difference (2.3% and 8.8%; *p*<0.0001), whereas that of NERD shows no difference (18.2% and 20.8%; *p* = 0.064).

**Conclusions:**

Significantly associated factors of NERD are considerably different from those of RE, indicating that these two disorders are pathophysiologically distinct. Eradication of *Helicobacter pylori* may have disadvantageous effects on RE but not on NERD.

## Introduction

Gastroesophageal reflux disease (GERD) is defined as the condition where reflux of stomach contents causes troublesome symptoms and/or complications [1]. The typical symptoms of GERD patients are heartburn and regurgitation [2], but other diverse symptoms can occur, including extraesophageal syndromes [1]. GERD is a frequent disease worldwide; even in Asia, known to have a lower rate of incidence, the prevalence of GERD has been reported as more than 5% [3]. Despite the high morbidity rate, the number of GERD patients has increased in these few decades [4], [5]. For example, the prevalence of GERD in Eastern Asia was found to be 2.5–4.8% before 2005 and 5.2–8.5% from 2005 to 2010 [6]. As a very common disorder affecting millions of people across the globe, it is important to clarify the etiology and pathogenesis of GERD.

Though the reflux of intragastric contents is defined as the etiology, the underlying mechanism of GERD has not been adequately elucidated. Multiple factors have been reported to be associated with GERD such as age [3], [7], gender [3], [7], body mass index (BMI) [8], body weight [9], alcohol drinking [3], [7], smoking [3], [7], etc., but past studies have shown conflicting results. One of the reasons for difficulty in identifying causative factors for GERD is the confusion between reflux esophagitis (RE, diagnosed by endoscopic observation) and non-erosive reflux disease (NERD, mainly diagnosed on the basis of the upper gastrointestinal symptoms) [1]. Since both disorders present different clinical features, we are convinced that the definitions of RE and NERD should be strictly separated. In our present study, therefore, RE and NERD patients were stringently separated before analyses, based upon endoscopic observation and detailed questionnaires.

We are also convinced that precise evaluation of the adjacent stomach state is necessary, since reflux of gastric acid is the main cause of not only RE but also NERD. It is well known that chronic *Helicobacter pylori* (*H. pylori*) infection induces gastric atrophy, which then leads to a lower gastric acid secretion [10], [11]. Probably reflecting the hypoacidity accompanied with atrophic change of gastric mucosa, presence of *H. pylori* infection has been reported to be one of the protective factors for GERD[7], [12]–[14]. In our present study, we precisely evaluated not only *H. pylori* infection state, but also the degree of gastric atrophy. As indicators of gastric atrophy, we assessed serum levels of pepsinogen I (PG I, produced by the chief and mucous neck cells in the fundic glands) and pepsinogen II (PG II, produced by not only the chief and mucous neck cells in the fundic glands but also by the cells in the pyloric glands and Brunner's glands). The progression of gastric atrophy leads to a gradual decrease in the level of PG I while the level of PG II remains fairly constant [15]. As a result, PG I/II ratio is a useful marker for evaluating the degree of gastric atrophy [16].

In our present study of more than 10,000 healthy subjects in Japan, we analyzed RE and NERD patients independently, compared with GERD-free subjects. Through the univariate and multivariate cross-sectional analyses of the large-scale healthy population, our study should shed light on the etiology and pathophysiology of both RE and NERD. In addition, to our knowledge, this is at present the largest study analyzing GERD in Asia. Therefore we are convinced that our study can provide the latest prevalence and correlated factors of RE and NERD in East Asia, the data of which is usually poor compared with Western countries.

## Methods

### Subjects

The study population was 20,773 subjects who received general medical checkup at Kameda Medical Center Makuhari (Chiba-shi, Chiba, Japan) from January 2010 to December 2010. All participants were over 20 years of age, and the former data were used in the case of having medical checkup twice in 2010. Criteria for exclusion were insufficient data (on age, sex, height, weight or laboratory data), history of upper gastrointestinal surgery, or intake of gastric acid inhibitors (histamine H_2_-receptor antagonists or proton pomp inhibitors). This study was approved by the ethics committees of the University of Tokyo, and written informed consent was obtained from each subject according to the Declaration of Helsinki.

### Evaluation of Serum anti-*H. pylori* Antibody and Serum Pepsinogen Levels

Serum anti-*H. pylori* IgG antibody was measured using a commercial EIA kit (E-plate “EIKEN” *Helicobacter pylori* antibody, Eiken Chemical Co LTD., Tokyo, Japan); antibody titer above a cut-off level of 10 U/ml was considered *H. pylori*-positive. Serum pepsinogen (PG) I and II were measured using a commercial RIA kit (E-Plate “EIKEN” Pepsinogen I and II, Eiken Chemical Co LTD.). In accordance with previous reports [17], [18], PG I levels were classified into ≤70 ng/ml and >70 ng/ml, and PG I/II ratio (PG I [ng/ml]/PG II [ng/ml]) were classified into ≤3 and >3.

### Age, Body Mass Index (BMI), and Questionnaire

For age, all subjects were categorized into nine age groups: <35, 35 to 39, 40 to 44, 45 to 49, 50 to 54, 55 to 59, 60 to 64, 65 to 69, and ≥70 (20–39: younger age, 40–59: middle age, and ≥60 years: older age). For BMI, all subjects were categorized into three subgroups: <18.5 (underweight), 18.5 to 25.0 (normal range), and ≥25.0 (overweight), according to the World Health Organization classification of BMI [19].

A detailed questionnaire inquiring about symptoms related to the upper gastrointestinal tract, medical history, family history, lifestyle factors, etc. was completed by every participant. For alcohol intake, the subjects were classified into presence (drink sometimes, drink almost everyday, or drink everyday) or absence (never drink or rarely drink) of drinking habit. For smoking, each subject was classified as a current smoker or a current nonsmoker (including subjects with history of smoking).

### Definition of Endoscopic Reflux Esophagitis (RE) and Non-erosive Reflux Disease (NERD)

By endoscopy, all the study subjects were diagnosed as RE(+) or RE(−) subjects according to the Los Angeles classification [20]. RE was defined as the presence of mucosal breaks: i.e., grade LA-A, -B, -C, or -D based on the Los Angeles classification.

Diagnosis of NERD is much more difficult, since it depends mainly on the assessment of symptoms. Various questionnaires and methods evaluating GERD and NERD have been proposed, such as proton pump inhibitor test [21], pH monitoring [21], QUEST [22], FSSG (Frequency Scale for the Symptoms of GERD) [23], etc. Although many indexes exist, we decided to simply focus on the presence of heartburn and acid regurgitation above-mentioned, as both symptoms are thought to be cardinal symptoms of GERD [1], [2]. We picked two questions for heartburn and acid regurgitation: “Do you get heartburn?” and “Do you get bitter liquid (gastric acid) coming up into your throat?”, answers of which were respectively selected from “always”, “often”, “sometimes”, “occasionally”, and “never” [24]. The presence of heartburn and acid regurgitation was defined as “often” or “always” having the symptom. Based on evaluating the frequency of symptoms, NERD was defined as the presence of heartburn and/or acid regurgitation in the RE-free subjects.

### Statistical Methods

In the univariate analyses, chi-square test was used for evaluating differences between RE patients and GERD-free subjects, and also for evaluating differences between NERD patients and GERD-free subjects. Multiple logistic regression model was next applied for assessing predictive background factors selected from the univariate analyses. A two-sided *p* value of less than 0.05 was considered statistically significant. All statistical analyses were performed using the JMP 9.0 or SAS 9.1.3 software (SAS Institute Inc., Cary, NC, USA).

## Results

### Baseline Characteristics of Study Subjects

**Figure 1:**
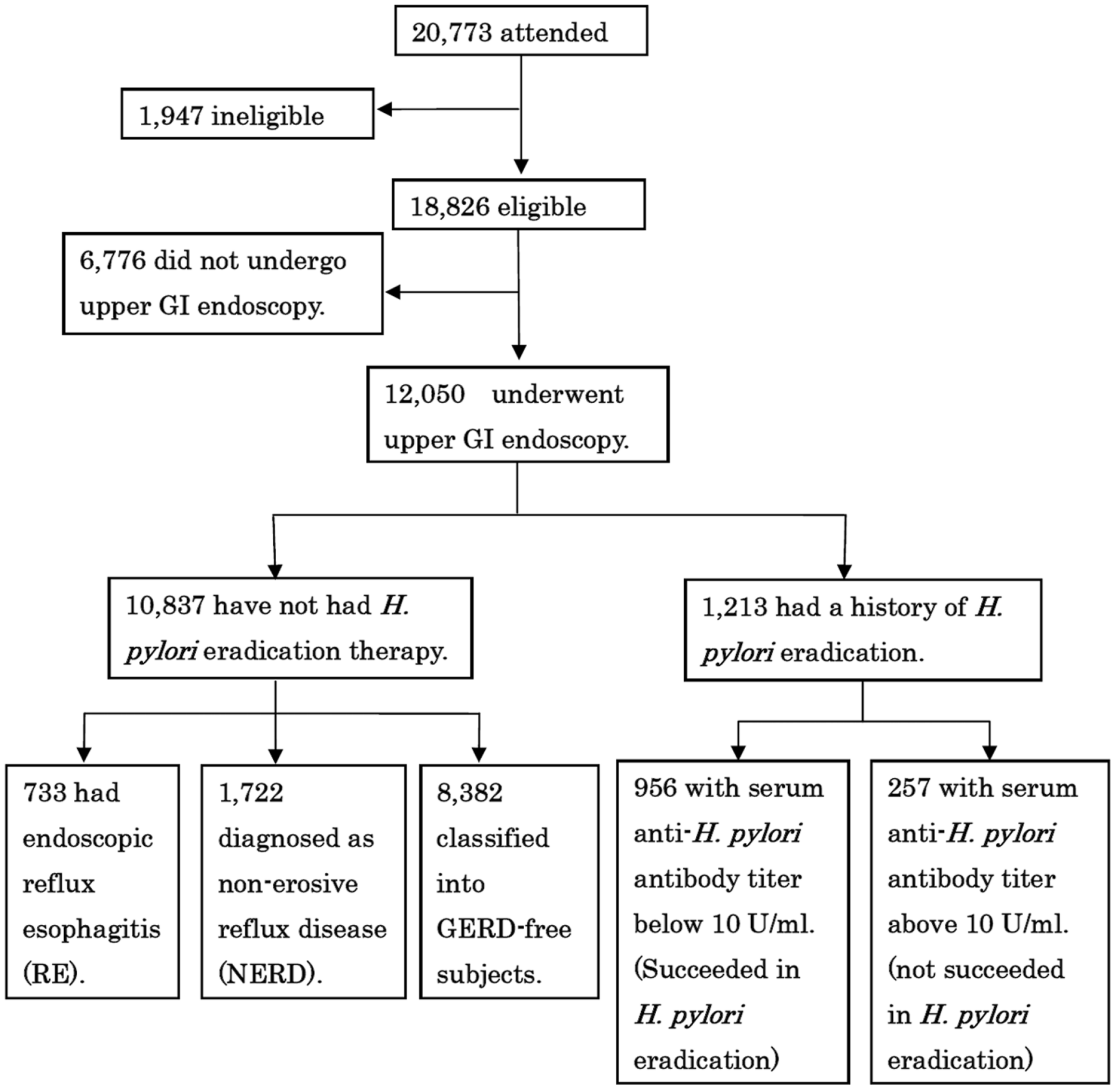
Study recruitment flowchart. Among the 20,773 subjects who attended this study, we excluded 1,928 subjects as follows; 820 subjects with insufficient data (lack of age, sex or laboratory data), 210 subjects with a history of upper gastrointestinal surgery, and 898 subjects taking gastric acid suppressant (H_2_-receptor antagonists or proton pomp inhibitor). GI, gastrointestinal; *H. pylori*, *Helicobacter pylori*; RE, reflux esophagitis; NERD, non-erosive reflux disease.

Among the 20,773 subjects who attended our study, we excluded 1,947 subjects because of insufficient data, a history of upper gastrointestinal surgery or intake of gastric acid suppressants (proton pomp inhibitor and/or H_2_-receptor antagonist). Of the 18,826 eligible subjects, 12,050 underwent upper gastrointestinal (GI) endoscopy (Figure 1). 1,213 were further excluded because of history of *H. pylori* eradication, leading to a final study population of 10,837 subjects which comprised of 6,332 men and 4,505 women with a mean age of 50.9±9.4 years (range 20–87 years). Of the 10,837 study subjects, 733 (6.8%) presented endoscopic reflux esophagitis (RE) and 1,722 (15.9%) were diagnosed as non-erosive reflux disease (NERD).

### Associated Factors for Endoscopic Reflux Esophagitis (RE)

The characteristics of participants based on the presence of RE is shown in Table 1. Univariate analyses were carried out with several putative factors which had been reported to be positively correlated with RE: male gender [25], [26], higher BMI or obesity [12], [27], [28], older age [12], alcohol consumption [25], [29], and so on. We also evaluated chronic *H. pylori* infection and gastric atrophy, which in contrast have been reported as negative risk factors of RE[25], [29]–[32].

**Table 1:** Comparison between endoscopic reflux esophagitis (RE) patients and GERD-free subjects.

Factors	RE patients (n = 733)	GERD-free subjects (n = 8,382)	Odds Ratio (95% CI)	*p* value
**Age (years)**	24–76 (50.9±8.8) y.o.	20–87 (51.0±9.4) y.o.		0.2465
** 35>**	15 (2.0%)	237 (2.8%)	reference	
** 35–39**	64 (8.7%)	757 (9.0%)	1.3 (0.84–2.10)	
** 40–44**	103 (14.1%)	1,240 (14.8%)	1.6 (1.02–2.44)	
** 45–49**	134 (18.3%)	1,351 (16.1%)	1.8 (1.19–2.83)	
** 50–54**	151 (20.6%)	1,605 (19.1%)	1.7 (1.10–2.62)	
** 55–59**	152 (20.7%)	1,651 (19.7%)	1.5 (0.98–2.34)	
** 60–64**	77 (10.5%)	978 (11.7%)	1.3 (0.80–1.98)	
** 65–69**	20 (2.7%)	361 (4.3%)	1.2 (0.70–1.97)	
** ≥70**	17 (2.3%)	202 (2.4%)	1.5 (0.83–2.55)	
**Gender**				<0.001*
** Male**	623 (85.0%)	4,756 (56.7%)	reference	
** Female**	110 (15.0%)	3,626 (43.3%)	0.2 (0.19–0.28)	
***H. pylori***				<0.001*
*** H. pylori*** ** (−)**	653 (89.1%)	5,582 (66.6%)	reference	
*** H. pylori*** ** (+)**	80 (10.9%)	2,800 (33.4%)	0.2 (0.19–0.31)	
**PG I**				0.332
** PG I >70**	124 (16.9%)	1,303 (15.5%)	reference	
** PG I ≤70**	609 (83.1%)	7,079 (84.5%)	0.9 (0.74–1.11)	
**PG I/II ratio**				<0.001*
** PG I/II >3**	710 (96.9%)	6,716 (80.1%)	reference	
** PG I/II ≤3**	23 (3.1%)	1,666 (19.9%)	0.1 (0.09–0.20)	
**BMI**	15.4–39.1 (24.8±3.4)	13.7–48.3 (22.8±3.2)		<0.001*
** 18.5>**	13 (1.8%)	528 (6.3%)	reference	
** 18.5–24.9**	404 (55.1%)	6,062 (72.3%)	2.7 (1.55–4.74)	
** 25.0≤**	316 (43.1%)	1,792 (21.4%)	7.2 (4.08–12.58)	
**Alcohol**				<0.001*
** Nondrinker**	188 (25.6%)	3,376 (40.3%)	reference	
** Drinker**	545 (74.4%)	5,006 (59.7%)	2.0 (1.65–2.32)	
**Smoking**				<0.001*
** Nonsmoker**	219 (29.9%)	6,899 (82.3%)	reference	
** Smoker**	514 (70.1%)	1,483 (17.7%)	2.0 (1.68–2.34)	

CI, confidence interval; *H. pylori*, *Helicobacter pylori*; PG, pepsinogen; BMI, body mass index; y.o., years old. Chi-square test was used for statistical evaluation, and the correlation of each background factor between RE patients and GERD-free subjects was calculated respectively. The level of significance in the univariate analyses was set at *p* value <0.05 (*).

In our large-scale study of Japanese population, univariate analyses demonstrated that gender, *H. pylori* infection, BMI, PG I/II ratio, alcohol intake, and smoking are statistically significant factors for RE (Table 1). Similar to previous studies [12], older age showed a positive correlation with RE, although there was no linear trend between age and RE through the detailed analyses. Our study showed that the highest odds ratio and frequency of RE patients was not in the older age ranges but in the middle age ranges (from 45 to 59 years, Figure S1).

Based on the univariate analyses (Table 1), we excluded non-significant PG I but did not exclude age as a basic factor. The following multivariate analysis showed that RE was positively correlated with male gender, *H. pylori* non-infection, higher PG I/II ratio, higher BMI, alcohol drinking, older age, and smoking (Table 2). The correlations of selected seven factors were mostly consistent with previous reports[12], [25]–[30], [32]. In more detailed analyses using categorized age group (Table S1), middle-aged participants had a stronger association with RE, similar to the abovementioned univariate analysis (Table 1). More detailed analysis using categorized BMI also showed that the value of BMI and the risk for RE are positively correlated (Table S1).

**Table 2:** Correlation between endoscopic reflux esophagitis (RE) and selected background factors.

Variables	Standardized coefficients	Odds Ratio (95% CI)	*p* value (<0.05)
**Gender (reference: male)**	−0.557	0.57 (0.51–0.64)	<0.001*
***H. pylori*** ** (reference: negative)**	−0.552	0.58 (0.51–0.65)	<0.001*
**PG I/II ratio (reference: PG I/II>3)**	−0.496	0.61 (0.50–0.72)	<0.001*
**BMI**	0.464	1.60 (1.48–1.71)	<0.001*
**Alcohol (reference: nondrinker)**	0.161	1.17 (1.07–1.29)	<0.001*
**Age**	0.148	1.16 (1.07–1.26)	<0.001*
**Smoking (reference: nonsmoker)**	0.129	1.14 (1.06–1.29)	<0.001*

CI, confidence interval; *H. pylori*, *Helicobacter pylori*; PG, pepsinogen; BMI, body mass index. We evaluated age and BMI as continuous variables. Multiple logistic regression analysis was applied to calculate standardized coefficients and odds ratio for selected seven variables. The seven variables are shown in order of the absolute values of standardized coefficients. The level of significance was set at *p* value <0.05 (*).

### Associated Factors for Non-erosive Reflux Disease (NERD)

NERD is defined as the presence of troublesome GERD symptoms (heartburn and/or acid regurgitation) with no endoscopically-visible damage of the esophageal mucosa. Similar to many recent studies [33],[34], the number of NERD patients (15.9%) is larger than that of RE patients (6.8%) in our cohort. The positive and negative risk factors for NERD remain controversial, although the association of NERD with many predictive factors such as gender [12],[33],[35],[36], BMI [12],[33], age [33], smoking [12],[33], alcohol [12], *H. pylori* infection [12],[33],[36], etc. have been reported. In the past reports on NERD, however, the numbers of study subjects were rather small, and the background factors were mostly compared between NERD patients and RE patients [12],[33]. In our study, putative factors were analyzed focusing on the difference between NERD patients and GERD-free subjects, using a large cohort of more than 10,000 subjects.

Among the eight factors univariately analyzed (Table 3), age, PG I/II ratio, BMI, alcohol drinking, and smoking were statistically significant for NERD. The prevalence of NERD using categorized age groups suggested that the younger subjects tend to be suffering from NERD (Figure S2). The multivariate analysis using the same variables in Table 2 was next performed, which showed that *H. pylori* infection, female gender, higher PG I/II ratio, younger age, smoking, higher BMI, and alcohol drinking are positively associated factors for NERD (Table 4). For age, gender, and *H. pylori* infection, the directions of correlation for NERD were opposite to those for RE (Table 2 and 4), indicating that NERD is an utterly different disorder from RE. In addition, standardized coefficients of the predictive factors for NERD are much smaller than those for RE (Table 2 and 4), suggesting that the mechanism of NERD has not been sufficiently elucidated.

**Table 3:** Comparison between non-erosive reflux disease (NERD) patients and GERD-free subjects.

Factors	NERD patients (n = 1,722)	GERD-free subjects (n = 8,382)	Odds Ratio (95% CI)	*p* value (<0.05)
**Age (years)**	21–79 (50.1±9.2) y.o.	20–87 (51.0±9.4) y.o.		0.005*
** 35>**	45 (2.6%)	237 (2.8%)	reference	
** 35–39**	182 (10.6%)	757 (9.0%)	1.3 (0.89–1.81)	
** 40–44**	281 (16.3%)	1,240 (14.8%)	1.2 (0.85–1.68)	
** 45–49**	314 (18.2%)	1,351 (16.1%)	1.2 (0.87–1.72)	
** 50–54**	338 (19.6%)	1,605 (19.2%)	1.1 (0.79–1.56)	
** 55–59**	287 (16.7%)	1,651 (19.7%)	0.9 (0.65–1.29)	
** 60–64**	174 (10.1%)	978 (11.7%)	0.9 (0.66–1.34)	
** 65–69**	65 (3.8%)	361 (4.3%)	0.9 (0.63–1.43)	
** ≥70**	36 (2.1%)	202 (2.4%)	0.9 (0.58–1.51)	
**Gender**				0.287
** Male**	953 (55.3%)	4,756 (56.7%)	reference	
** Female**	769 (44.7%)	3,626 (43.3%)	1.1 (0.95–1.17)	
***H. pylori***				0.236
*** H. pylori*** ** (−)**	1130 (65.6%)	5,582 (66.6%)	reference	
*** H. pylori*** ** (+)**	592 (34.4%)	2,800 (33.4%)	1.0 (0.94–1.17)	
**PG I**				0.272
** PG I >70**	286 (16.6%)	1,303 (15.6%)	reference	
** PG I ≤70**	1,436 (83.4%)	7,079 (84.4%)	0.9 (0.80–1.06)	
**PG I/II ratio**				0.013*
** PG I/II >3**	1,424 (82.7%)	6,716 (80.1%)	reference	
** PG I/II ≤3**	298 (17.3%)	1,666 (19.9%)	0.8 (0.74–0.97)	
**BMI**	14.4–42.7 (22.8±3.3)	13.7–48.3 (22.8±3.2)		0.011*
** 18.5>**	113 (6.6%)	528 (6.3%)	reference	
** 18.5–24.9**	1,186 (68.9%)	6,062 (72.3%)	0.9 (0.74–1.13)	
** 25.0≤**	423 (24.6%)	1,792 (21.4%)	1.1 (0.88–1.39)	
**Alcohol**				0.020*
** Nondrinker**	642 (37.3%)	3,376 (40.3%)	reference	
** Drinker**	1080 (62.7%)	5,006 (59.7%)	1.1 (1.02–1.26)	
**Smoking**				0.001*
** Nonsmoker**	1,360 (79.0%)	6,899 (82.3%)	reference	
** Smoker**	362 (21.0%)	1,483 (17.7%)	1.2 (1.04–1.37)	

CI, confidence interval; *H. pylori*, *Helicobacter pylori*; PG, pepsinogen; BMI, body mass index; y.o., years old. Chi-square test was used for statistical evaluation; the correlation of each subject background factor between NERD patients and GERD-free subjects was calculated respectively as an odds ratio (OR) with 95% confidence interval (CI). The level of significance in the univariate analyses was set at *p* value <0.05 (*).

**Table 4:** Correlation between non-erosive reflux esophagitis (NERD) and selected background factors.

Variables	Standardized coefficients	Odds Ratio (95% CI)	*p* value (<0.05)
***H. pylori*** ** (reference: negative)**	0.106	1.11 (1.04–1.19)	0.002†
**Gender (reference: male)**	0.099	1.10 (1.04–1.17)	0.001†
**PG I/II ratio (reference: PG I/II>3)**	−0.099	0.91 (0.85–0.97)	0.004†
**Age**	−0.099	0.91 (0.86–0.96)	<0.001†
**Smoking (reference: nonsmoker)**	0.080	1.08 (1.03–1.14)	0.003†
**BMI**	0.078	1.08 (1.03–1.14)	0.004†
**Alcohol (reference: nondrinker)**	0.076	1.08 (1.02–1.14)	0.008†

CI, confidence interval; *H. pylori*, *Helicobacter pylori*; PG, pepsinogen; BMI, body mass index. We evaluated age and BMI as continuous variables. Multiple logistic regression analysis was applied to calculate standardized coefficients and odds ratio for selected seven variables. The level of significance was set at *p* value <0.05 (†). The seven variables are shown in order of the absolute values of standardized coefficients.

The more minute multivariate analysis using categorized age groups and BMI was further performed (Table S2). For age, the younger subjects tend to have stronger association with NERD (Table S2), consistent with the result in Table 4. For BMI, it is also suggested that underweight (18.5>) or overweight (25.0≤) subjects are more likely to have NERD compared to subjects with standard BMI, although the correlation between BMI and NERD was not statistically significant (Table S2).

### Effects of *Helicobacter pylori* Eradication on GERD Patients

Most of previous studies showed negative association of RE with both *H. pylori* infection and atrophic gastritis [25],[30],[32]. On the other hand, association of NERD with *H. pylori* infection or atrophic gastritis is still controversial[33],[36]–[38]. As *H. pylori* infection is significantly associated with both RE and NERD in the present study (Table 2 and 4), we further analyzed the effects of *H. pylori* eradication on GERD patients. Judging from previous studies, the effect of *H. pylori* eradication on RE and NERD is also a disputable matter [39],[40]. We analyzed two groups: 3,472 subjects with chronic *H. pylori* infection (positive for serum *H. pylori* antibody without history of eradication therapy) and 956 subjects who succeeded in *H. pylori* eradication (negative for serum *H. pylori* antibody with history of eradication therapy).

The prevalence rate of RE was 2.3% among “chronic *H. pylori* infection” group and 8.8% among “*H. pylori* successfully eradicated” group (Table 5); there was a significant difference between the two groups (*p*<0.0001). On the other hand, the prevalence rate of NERD was 18.2% among “chronic *H. pylori* infection” group and 20.8% among “*H. pylori* successfully eradicated” group (Table 5); there was no statistical difference between the two groups (*p* = 0.064). From these results, it can be imagined that *H. pylori* eradication may have a disadvantageous effect on RE but may not affect NERD. Eradication of *H. pylori* may lead to occurrence or progression of reflux esophagitis (RE), which should be taken into consideration before executing *H. pylori* eradication therapy.

**Table 5:** Prevalence of reflux esophagitis (RE) and non-erosive reflux esophagitis (NERD) among “chronic *H. pylori* infection” subjects and “*H. pylori* successfully eradicated” subjects.

	Chronic *H. pylori* infectiongroup (n = 3,472)	*H. pylori* successfully eradicatedgroup (n = 956)	*p* value
**RE patients**	80 (2.3%)	84 (8.8%)	<0.0001†
**RE-free subjects**	3,392 (97.7%)	872 (91.2%)	
**NERD patients**	631 (18.2%)	199 (20.8%)	0.064
**NERD-free subjects**	2,841 (81.8%)	757 (79.2%)	

*H. pylori*, *Helicobacter pylori*. From the viewpoint of both RE and NERD, chi-square test was used for statistical evaluation. The level of significance in the univariate analyses was set at *p* value <0.05 (†).

## Discussion

### Prevalence and Trend of Reflux Esophagitis (RE) and Non-erosive Reflux Disease (NERD) in Japanese Healthy Population

GERD is more common in Western countries than in Asian countries [3]. For example, the prevalence of RE/NERD was 7.1/10.9% in Japan [26] or 8.0/4.0% in Korea [37], both of which were obviously lower than 15.5/27.1% in Sweden [35]. The background factors responsible for this marked difference have been believed to be racial characters [41], dissimilar BMI and physique [42], varied types and infestation of *H. pylori* [43], and so forth. In our study population of 10,837 Japanese subjects, however, the prevalence of RE and NERD was 6.8% and 15.9% respectively. We have found that the prevalence of GERD was much higher than those in previous Asian reports [26],[37], mostly due to increased numbers of NERD patients. We do not know whether the prevalence of GERD will reach similar level to Western countries in the future, but it is certain that disease rate of GERD is still increasing in Japan, probably based on a radical decrease of *H. pylori* morbidity and a fundamental change of lifestyle in the past few decades.

### Different Background Factors for Reflux Esophagitis (RE) and Non-erosive Reflux Disease (NERD)

Reflux of the stomach content, especially gastric acid, has been believed to be the main cause of both RE and NERD [1],[34]. Nevertheless, many studies including our present one have demonstrated that significantly associated factors for RE and NERD are considerably different.

First, our results showed that men are more likely to develop RE (men: 9.8%, women: 2.4%) whereas women are more likely to develop NERD (men: 15.1%, women: 17.1%). Contrastive sex difference of RE and NERD was also demonstrated by multivariate analyses (Table 2 and 4), which is consistent with many previous studies [25],[26],[35],[37],[44].

Second, our result showed that the association of age for RE and NERD presented the opposite tendency (Table 2 and 4, Figure S1 and S2). Most previous studies reported that the prevalence of RE increases with age [45],[46], but an association between NERD and age is still controversial. Our result is similar to Pilotto’s one, denoting a decreasing prevalence of NERD in association with age [46], though bimodal age distribution of NERD risk [37] or no association between NERD and age [35] have been also reported.

Third, like most previous reports [27],[44],[47], our results showed that increasing BMI is positively correlated with the incidence of RE. Conversely, the association between NERD and BMI has been still controversial; some studies have denoted that higher BMI is associated with NERD [44], whereas other studies have shown negative relations between BMI and NERD [12],[37]. Marginal association between BMI and NERD was detected in our analysis, although it was very weak in comparison with that between BMI and RE (Table 4).

Fourth, most previous studies have shown positive correlations between RE and drinking as well as between RE and smoking [12],[25],[29],[44], both of which were reconfirmed in our analysis (Table 2). Meanwhile, association of NERD with drinking and smoking has been also disputable [12],[37],[44]. Our analyses showed a positive correlation between smoking and drinking with not only RE but also NERD (Table 2 and 4), although the latter correlation is much smaller than the former one.

Marked background difference of RE and NERD should be due to their pathophysiology. RE, which for the most part is respondent to gastric acid suppressant [36], is thought to be mainly caused by excess of esophageal acid exposure. In contrast, the main mechanism behind NERD still remains unclear. Several mechanisms such as incomplete acid suppression, esophageal sensitivity to acid, abnormal tissue resistance, sustained esophageal contractions, etc. have been proposed [7],[34],[36], but we believe there exists an essential mechanism that has not yet been discovered.

### Study Limitations

First limitation of our study is that our study subjects were participants of medical checkup. We could not know whether the subjects with various GERD symptoms were certainly suffering from these symptoms and had need of medical treatment. Second limitation is our use of a questionnaire to define GERD symptoms. Esophageal impedance-pH monitoring test or PPI test was not undergone in this study. Therefore, true NERD was not rigorously distinguished from acid hypersensitive esophagus, non-acid hypersensitive esophagus, or functional heartburn [48],[49].

### Future Prospects

We are following the present cohort to verify identified factors in this study; the large-scale prospective analyses should help us confirm the definite risk factors. A time trend survey for GERD prevalence in Japan will also be anticipated. In particular, we plan to evaluate the influence of *H. pylori* eradication stringently, as the present cross-sectional analysis suggested that *H. pylori* eradication may have an unfavorable effect on RE, but not on NERD (Table 5). Many of the “chronic *H. pylori* infection” participants in this study have undergone *H. pylori* eradication therapy. Therefore, the effect of *H. pylori* eradication on not only GERD patients but also GERD-free healthy subjects should become clear in our next report, which is now still controversial [50].

In our study, NERD was defined as the presence of heartburn and/or acid regurgitation in RE-free subjects. These two are considered as the most typical GERD symptoms worldwide [1], but it goes without saying that there may be other patients who suffer other atypical symptoms. How to evaluate NERD is a fundamental problem that needs further assessment, especially in view of extraesophageal syndromes such as chronic cough, globus sensation, hoarseness, asthma, bronchitis, pneumonia, and so on [22],[24],[51],[52]. We have already tried to evaluate the multiple GERD symptoms in the large-scale study with 19,864 subjects [23], but in the future, more detailed questionnaire for evaluating the GERD symptoms should be performed in the larger-scale global cohort.

## Supporting Information

Figure S1 **Prevalence of reflux esophagitis (RE) patients in each age group among the 10,837 study subjects.** The histogram shows percentages of reflux esophagitis (RE) patients in nine age groups are shown.
(TIF)

Figure S2 **Prevalence of non-erosive reflux disease (NERD) patients in each age group among the 10,837 study subjects.** The histogram shows percentages of non-erosive reflux disease (NERD) patients in nine age groups.
(TIF)

Table S1 **Correlation between reflux esophagitis (RE) and seven selected factors based on age and BMI categorization.** CI, confidence interval; H. pylori, Helicobacter pylori; PG, pepsinogen; BMI, body mass index. Multiple logistic regression analysis was applied to calculate standardized coefficients and odds ratio for selected seven variables. The levels of significance in the multivariate analyses were set at p value <0.05 (*).
(DOC)

Table S2 **Correlation between non-erosive reflux esophagitis (NERD) and seven selected factors based on age and BMI categorization.** CI, confidence interval; H. pylori, Helicobacter pylori; PG, pepsinogen; BMI, body mass index. Multiple logistic regression analysis was applied to calculate standardized coefficients and odds ratio for selected seven variables. The level of significance in the multivariate analyses was set at p value <0.05 (*).
(DOC)
