# Supplementary figures and images for: Background Factors of Reflux Esophagitis and Non-Erosive Reflux Disease: A Cross-Sectional Study of 10,837 Subjects in Japan
Source: PLoS One. 2013 Jul 26;8(7):e69891. doi: 10.1371/journal.pone.0069891 (PMC3724738; doi:10.1371/journal.pone.0069891)

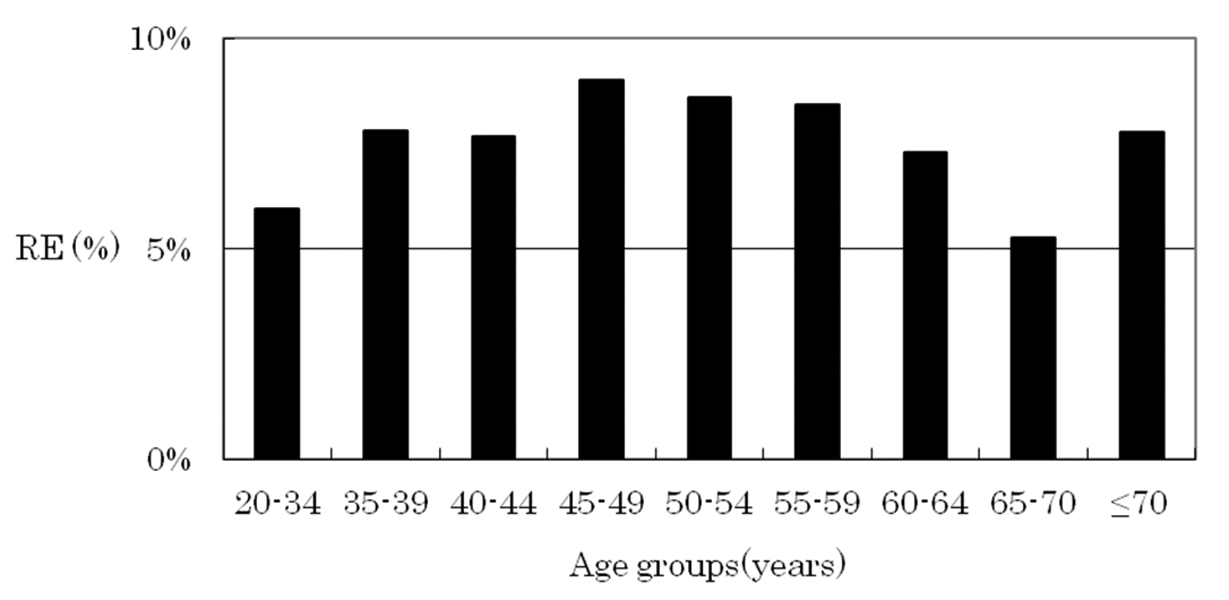

Supplement: Figure S1 — Prevalence of reflux esophagitis (RE) patients in each age group among the 10,837 study subjects. The histogram shows percentages of reflux esophagitis (RE) patients in nine age groups are shown. (TIF) [file pone.0069891.s001.tif]

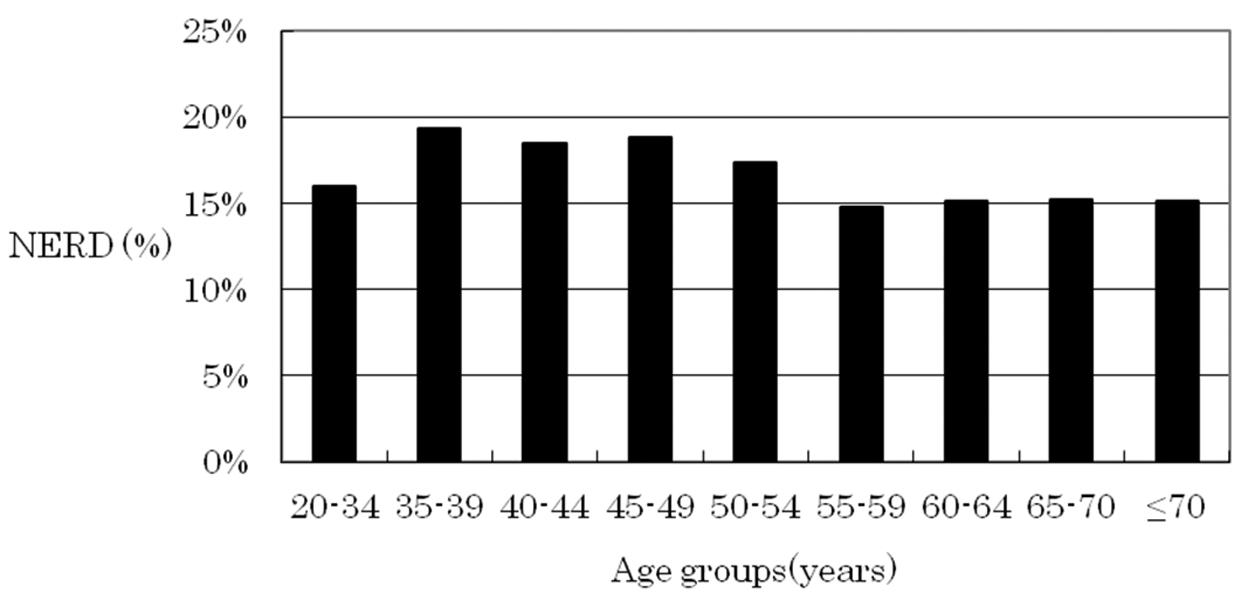

Supplement: Figure S2 — Prevalence of non-erosive reflux disease (NERD) patients in each age group among the 10,837 study subjects. The histogram shows percentages of non-erosive reflux disease (NERD) patients in nine age groups. (TIF) [file pone.0069891.s002.tif]
