# Supplementary material for: Background Factors of Reflux Esophagitis and Non-Erosive Reflux Disease: A Cross-Sectional Study of 10,837 Subjects in Japan
Source: PLoS One. 2013 Jul 26;8(7):e69891. doi: 10.1371/journal.pone.0069891 (PMC3724738; doi:10.1371/journal.pone.0069891)
Supplement: Table S2 — Correlation between non-erosive reflux esophagitis (NERD) and seven selected factors based on age and BMI categorization. CI, confidence interval; H. pylori, Helicobacter pylori; PG, pepsinogen; BMI, body mass index. Multiple logistic regression analysis was applied to calculate standardized coefficients and odds ratio for selected seven variables. The level of significance in the multivariate analyses was set at p value <0.05 (*). (DOC) [file pone.0069891.s004.doc]

Table S2. Correlation between non-erosive reflux esophagitis (NERD) and seven selected factors based on age and BMI categorization.

| **Variables** | **Standardized coefficients** | **Odds Ratio**  **(95% CI)** | ***p* value** |
| --- | --- | --- | --- |
| **Age (reference: 20-34)** |  |  |  |
| **35-39** | 0.065 | 1.07 (0.96-1.19) | 0.221 |
| **40-44** | 0.059 | 1.06 (0.94-1.20) | 0.351 |
| **45-49** | 0.066 | 1.07 (0.94-1.22) | 0.309 |
| **50-54** | 0.032 | 1.03 (0.91-1.19) | 0.645 |
| **55-59** | -0.040 | 0.96 (0.84-1.10) | 0.558 |
| **60-64** | -0.017 | 0.98 (0.88-1.11) | 0.769 |
| **65-69** | -0.003 | 1.00 (0.92-1.09) | 0.940 |
| **≥ 70** | -0.003 | 1.00 (0.93-1.07) | 0.933 |
| **Gender (reference: male)** | 0.091 | 1.10 (1.03-1.16) | 0.002* |
| ***H. pylori* (reference: negative)** | 0.109 | 1.12 (1.04-1.19) | 0.001* |
| **PG I/II ratio (reference: PG I/II>3)** | -0.099 | 0.91 (0.85-0.97) | 0.004* |
| **BMI (reference:** **≤18.5)** |  |  |  |
| **18.5-24.9** | -0.020 | 0.98 (0.89-1.08) | 0.692 |
| **25.0 ≤** | 0.064 | 1.07 (0.97-1.18) | 0.204 |
| **Alcohol (reference: Nondrinker)** | 0.075 | 1.08 (1.02-1.14) | 0.009* |
| **Smoking (reference: Nonsmoker)** | 0.082 | 1.09 (1.03-1.14) | 0.002* |

CI, confidence interval; *H. pylori*, *Helicobacter pylori* ; PG, pepsinogen; BMI, body mass index. Multiple logistic regression analysis was applied to calculate standardized coefficients and odds ratio for selected seven variables. The level of significance in the multivariate analyses was set at *p* value <0.05 (*).
